# Eucaine “B”—A Further Note on New Local Anesthetics

**Published:** 1897-06

**Authors:** J. C. Clemesha

**Affiliations:** 329 Franklin Street


### EUCAINE "B"—A FURTHER NOTE ON NEW LOCAL ANESTHETICS.

By J. C. CLEMESHA, M. D., L. R. C. P., London, M. R. C. S., England.

ONE evening in September of last year I read a short article entitled "Eucaine—a note on the new local anesthetic," before the surgical section of the Buffalo Academy of Medicine, describing the properties of the new discovery, and its advantages and disadvantages, with respect to cocaine in ophthalmic practice.

The most serious objection to the use of eucaine solutions lay in their irritating properties when instilled in the eye, the conjunctiva becoming markedly hyperemic and the patient complaining of a smarting, pricking sensation, lasting some minutes.

Gradually eucaine was less and less frequently used at the Buffalo Eye and Ear Infirmary, wholly, I believe, on account of the above-mentioned symptoms arising from its use, until at last it was employed merely to demonstrate its anesthetic properties to the medical students of the University of Buffalo attending Dr. Howe's clinic.

Lately, however, Professor Merling, of Berlin, has produced, synthetically, a new substance which, under the name of eucaine "B," claims to possess all the desirable properties of eucaine "A," minus its irritating and congestive quality.

Briefly stated eucaine "B" is the hydrochlorate of benzoylvinyldiacetonalkamine and closely related not only to the older eucaine "A," but also to cocaine, and especially to tropococaine. It is, however, less toxic than the latter drugs and its solutions are sterilised on boiling. It is soluble in warm water to the extent of 5 per cent. and, physiologically considered, its main difference from eucaine "A" is that its irritant effects are minimal.

Being interested to learn personally the action of this new substance, a 2 and 5 per cent. solution were procured and their action contrasted with similar solutions of cocaine. First, three drops of a eucaine "B" 2 per cent. solution were instilled in my right eye, and three drops of a cocaine 2 per cent. solution in my left. The eucaine "B" caused a passing, burning sensation, more noticeable than that produced by the cocaine, though there was an absence of that peculiar stiffness experienced in the cocainised eye. The degree of anesthesia produced was about alike in either eye, though it took longer for the eucaine "B" to bring about its effect, and while the pupil of left eye became dilated from the cocaine solution, that of the right eye remained stationary. Furthermore, there was no congestion of the conjunctiva, which would surely have followed the instillation of a 2 per cent. eucaine "A" solution.

After the preliminary experiment upon myself I employed the eucaine "B" solution in three lachrymal cases and noted that the patients remarked that the drops caused more smarting than the ones used on former occasions, *i. e.*, cocaine, but that it was merely transitory, a passing unpleasantness. The eucaine "B" solution produced a satisfactory anesthesia and slight, if any, congestion of the conjunctiva; the cornea remained clear and did not show that roughness or shriveled appearance seen after the application of cocaine.

Later, Dr. Howe did an iridectomy. In this case six drops of a 5 per cent. solution of eucaine "B," one drop at intervals of two minutes, produced a successful anesthetic state, although the patient claimed that she felt the operation more than she did a similar one on the other eye, performed under the effect of cocaine, but it must be stated that in the former iridectomy ten drops of a 5 per cent. cocaine solution were the means by which the state of local anesthesia was brought about. There was no noticeable irritation or injection of the conjunctiva and beyond a preliminary smarting no uncomfortable effects were noticed.

In three patients with steel imbedded in the cornea, a 2 per cent. solution of eucaine "B" was used as a preliminary measure to removing the foreign bodies. In all cases the anesthesia was satisfactory, though not induced as quickly as that from cocaine. There certainly was no dilatation of the pupils, inappreciable if any, hyperemia of the conjunctiva, and slight smarting when first instilled into the conjunctival cul-de-sac.

The following conclusions, I think, may be drawn from the above cases: Eucaine "B" is an efficient local anesthetic and may be substituted for cocaine. It causes slightly more smarting upon application and appears somewhat slower in producing anesthesia than cocaine. It does not dilate the pupil or cause cloudiness of the cornea as does cocaine. It does not irritate or cause conjunctival hyperemia, as does eucaine "A," but, like it, is sterilised by boiling and will keep for long periods.

As a result of some observations in nose and throat cases, Dr. Millener suggests that, while eucaine "B" produces a similar state of anesthesia as eucaine "A" and cocaine, it does not cause the irritation and congestion of the parts as does the former, though slower in its action than the latter. It is intensely bitter to the taste and not as soluble as eucaine "A," and whereas eucaine "A," when applied to the mucous membrane of the nasal cavities, causes a burning sensation and a slight watery discharge due to irritative properties, eucaine "B" does neither.

The hemorrhage resulting from a nasal operation, using eucaine "B," is not greater than one done under the effect of cocaine, though in the latter the hemorrhage may be delayed, due to the primary anemia of the parts, and perhaps the tendency towards hemorrhage is less, as the subsequent dilatation of the vessels, when cocaine is used, often causes profuse and prolonged bleeding, while in eucaine "B," though at first dilatation of the vessels is present, the secondary effect is that of constriction of the capillaries and cessation of the hemorrhage.

In a case of hypertrophic catarrh, he removed an hypertrophy of the lower turbinated on the left side, using a 5 per cent. eucaine "B" solution as the local anesthetic. Anesthesia was complete in six minutes. There was no apparent congestion of the parts and the bleeding, though severe for a minute or so, ceased quickly.

On the right side, same case, in removing a similar sized hypertrophy, eucaine "A" was used and it was noted that much congestion ensued on application of the solution, though the subsequent hemorrhage was not at all severe.

329 Franklin Street.

---

Serum Diagnosis of Typhoid Fever.—Delépine, of Manchester, (*Universal Medical Journal*) emphasised the importance of using fresh cultures of the bacillus typhosus ; he had noticed that old cultivations had of themselves a tendency to "clumping." To obtain such cultures the bacillus was grown on solid medium and fluid cultures were made from day to day as required. It was important to avoid the slightest contamination, and he considered a magnifying power of 300 diameters sufficient to observe the reaction.

